# Analysis of antibiotic consumption in burn patients

**DOI:** 10.3205/dgkh000252

**Published:** 2015-06-09

**Authors:** Somayeh Soleymanzadeh-Moghadam, Leila Azimi, Laleh Amani, Aida Rastegar Lari, Faranak Alinejad, Abdolaziz Rastegar Lari

**Affiliations:** 1 Department of Biology, University of Qom, Qom, Iran; 2 Burn Research Center, Iran University of Medical Sciences, Tehran, Iran; 3 Soins Infirmiers – CEGEP Limoilou, Québec, Canada

**Keywords:** antibiotic prescription, resistance, burn patients

## Abstract

Infection control is very important in burn care units, because burn wound infection is one of the main causes of morbidity and mortality among burn patients. Thus, the appropriate prescription of antibiotics can be helpful, but unreasonable prescription can have detrimental consequences, including greater expenses to patients and community alike.

The aim of this study was to determine the effect of antibiotic therapy on the emergence of antibiotic-resistant bacteria. 525 strains of *Pseudomonas aeruginosa, Acinetobacter baumannii* and *Staphylococcus aureus* were isolated from 335 hospitalized burn patients. Antibiotic susceptibility tests were performed after identification the strains. The records of patients were audited to find the antibiotic used.

The results indicated that *P. aeruginosa* is the most prevalent Gram-negative bacteria. Further, it showed a relation between abuse of antibiotics and emergence of antibiotic resistance. Control of resistance to antibiotics by appropriate prescription practices not only facilitates prevention of infection caused by multi-drug resistant (MDR) microorganisms, but it can also decrease the cost of treatment.

## Zusammenfassung

Infektionskontrolle ist wichtig in Verbrennungseinheiten, weil Infektionen eine der Hauptursachen der Morbidität und Mortalität bei Brandverletzten sind. Während die richtige Antibiotikaauswahl lebensrettend sein kann, kann die falsche Auswahl unerwünschte Folgen haben, verbunden mit erhöhten Kosten für den Patienten und für die Gesellschaft.

Zielsetzung der Studie war die Untersuchung des Einflusses der Antibiotikatherapie auf die Ausbreitung resistenter Bakterien. Hierzu wurde die Antibiotikaempfindlichkeit bei 525 *Pseudomonas aeruginosa-, Acinetobacter baumannii-* und *Staphylococcus aureus*-Stämmen untersucht, die von 335 Brandverletzten isoliert worden waren. Die eingesetzten Antibiotika wurden aus der Patientendokumentation entnommen.

Im Ergebnis war *P. aeruginosa* der am häufigsten isolierte Erreger. Es zeigte sich ein Zusammenhang zwischen Antibiotikamissbrauch und Antibiotikaresistenz. Die Bestimmung der Antibiotikaresistenz ist nicht nur entscheidend für den Therapieerfolg, sondern kann die Ausbreitung multiresistenter Bakterien eindämmen und die Behandlungskosten reduzieren.

## Introduction

Burns and subsequent consequences are a global problem [1]. Burn wound infection is one of the most common causes of death in burn injuries. Further, burn patients are at high risk for nosocomial infection [2]. Thus, infection control in burn patients especially in the first 5 days after hospitalization is important. Infection control during this time period can prevent morbidity and mortality in these patients [2]. Appropriate and accurate antibiotic prescription can be considered an important factor in increasing the awareness of patients about proper antibiotic use. Over the past years, the antibiotic consumption in Iran has increased [3]. The appropriate use of antibiotics can improve the health of burn patients, but inappropriate prescription and use of antibiotics can have deleterious consequences [3], e.g., it can result in antibiotic resistance which increases the cost of healthcare to both patients and the community [3], [4]. Globally, the cost of antibiotic therapy was estimated at approximately 40 billion dollars in 2000. It is notable that the proportion of developing countries increased from 19% in 1990 to 34% in 2000 [5]. For Iran, the total medical market sales reached $2.467 billion in 2010 [6], with an average annual growth of drug consumption of 11.5% [7], [8]. However, the average annual increase in drug consumption was 9% worldwide and 7% in developing countries. The high use of antibiotics in Iran ranks this country among the first 20 countries worldwide [7], [8]. Burn patients, especially severely injured ones, have a high risk of nosocomial infections. Due to rising antibiotic resistance worldwide, burn patients are at increasing risk of infection with MRSA as well as *P. aeruginosa *and* A. baumannii* [9], [10], [11]. This poses an important challenge to infection control. Therefore, it is necessary to implement appropriate antibiotic therapy protocols, especially in burn patients. 

## Materials and methods

### Setting

This cross-sectional study was conducted from August 2012 to March 2013. 685 specimens were collected from 335 wound infections of burn patients who were hospitalized in different wards at Motahary hospital, a tertiary burn care hospital in Tehran with 3 wards (for men, women, and children separately). 

### Bacterial strains 

Identification of isolates of *P. aeruginosa, A. baumannii *and* S. aureus* as the most important bacteria in nosocomial infection in burn patients in Iran [9], [10], [11] was performed by routine biochemical and microbiological tests. Patients’ information was collected from their records. 

### Antibiotic susceptibility testing

Antibiotic susceptibility testing of *P. aeruginosa *and *A. baumannii* was performed for ticarcillin (75 µg), piperacillin (100 µg), piperacillin-tazobactam (100/10 µg), ceftazidime (30 µg), cefotaxime (30 µg), cefepime (30 µg), aztreonam (30 µg), imipenem (10 µg), gentamicin (10 µg), tobramycin (10 µg), amikacin (30 µg), ciprofloxacin (5 µg), colistin (10 µg), tetracycline (30 µg), and trimethoprim/sulfamethoxazole (1.25/23.75), according to the CLSI guideline with standard antibiotic discs from MAST. 

Antibiotic susceptibility testing of *S. aureus* was performed for vancomycin (30 µg), rifampicin (5 µg), fusidic acid (10 µg), fosfomycin (200 µg), erythromycin (15 µg), cefotaxime (30 µg), cefepime (30 µg), trimethoprim/sulfamethoxazole (1.25/23.75), oxacillin (1 µg), and gentamicin (10 µg). Polimixina B and novobiocin discs were used for the identification of *S. aureus*. 

### Antibiotics prescribed 

To determine the consumption of antibiotics, patients’ records were reviewed and antibiotic prescription was assessed. Generally, 22 types of antibiotics were prescribed, from one up to 11 types for each patient. These antibiotics include amikacin, meropenem, vancomycin, colistin, cephalexin, ceftazidime, cefepime, clindamycin, cefexime, imipenem, gentamicin, tazocin, ciprofloxacin, cefazolin, ofloxacin, metronidazole, ampicillin, penicillin, fortamet, levofloxacin, nalidixic acid and targocid. 

### Statistical analysis 

Age, sex, burn percentage, cause of burn, duration of hospitalization, burn degree and rate of mortality of patients were analyzed using SPSS software, version 17.0 and Excel 2010. Chi-square, Mann-Whitney and Fisher’s exact tests were performed and p<0.05 was considered significant.

## Results

525 isolates of *P. aeruginosa, A. baumannii *and *S. aureus* were collected, comprising 295 (56.2%), 159 (30.4%) and 71 (13.5%) isolates, respectively. Table 1 (Tab. 1) and Table 2 (Tab. 2) show the different isolates’ resistance to various antibiotics in percent. According to patient records, a total of 22 types of antibiotics were prescribed for burn patients, and each patient consumed from one to 11 types of antibiotics (Table 3 (Tab. 3)). The types of antibiotics consumed are shown in Table 4 (Tab. 4). The relationship between type of antibiotic and results of antibiotic susceptibility testing was assessed in patients for whom we had both their antibiotic therapy and the results of antibiotic susceptibility testing (Table 5 (Tab. 5)). Chi-square and Fisher’s exact tests showed a significant relationship between the use of cefotaxime, ciprofloxacin and piperacillin-tazobactam and the development of resistance (p≤0.04).

## Discussion

The results of this study indicate that *P. aeruginosa* is the most prevalent Gram-negative bacterial species isolated from burn patients; *A. baumannii *and* S. aureus* were the second and third most prevalent species, resp., that caused wound infection. Other studies in 2011 and 2012 conducted in Iran confirmed our results [10], [11]. In this regard, the study conducted in Iran in 2013 indicated that the expanded use of beta-lactam antibiotics can not only select the beta-lactam resistant bacteria but also give rise to cross-resistance among different bacterial species [12]. Globally, antibiotics are among the best-selling drugs [6]. Data collected during first half of the year at the Emam University Hospital in Iran showed an average antibiotic consumption of 279 DDD (Defined Daily Doses)/ 100 patient days in ICUs [13]; in contrast, the use of antibiotics at German ICUs accounted for an average of 140 DDD/ 100 patient day [14]. According to our results, resistance to tested antibiotics in *A. baumannii* is significantly greater than in *P. aeruginosa*, except for colistin and tetracyclin (p≤0.05). Appropriate treatment can control disease progress and nosocomial infection. The results of this study indicated that treatment of patients with imipenem, meropenem, ciprofloxacin and aminoglycoside was effective in more than 80% of the cases, despite the observed resistance to these antibiotics. These records provide evidence of overuse of antibiotics and a lack of attention to laboratory results, which facilitates the spread of antibiotic resistance. The results of a study conducted in Thailand indicated that 26% of antibiotic prescriptions were incorrect [15]. In another study conducted in Saudi Arabia, it was shown that recognition and prescription of antibiotics was inappropriate in more than 50% of patients [16]. Similarly, in a study conducted in Iran, antibiotic prescription, the dose prescribed, and indications for antibiotic prescription were erroneous in 40% of the cases [17]. According to the results of the current study, beta-lactam antibiotics are the most frequently prescribed, and most antibiotic resistance was observed against this family of antibiotics in the tested isolates. For example, 70% of cefepime prescription and more than 90% and 98% cefepime resistance were observed in *P. aeruginosa *and* A. baumannii*, respectively. Patient records have shown that generally, antibiotic therapy in burn patients started with cefepime; this could be an important cause of a greater prevalence of cefepime resistance among burn patients. The rate of inapproprate antibiotic prescription has been cited as 41% to 91% worldwide, making it a global problem [1]. WHO policy is aimed at proper use of antibiotics to treat infection while minimizing side-effects and expense. Moreover, attention to dose and duration of antibiotic treatment is considered in this policy [18].

## Conclusion

One of the most urgent concerns in the medical community today is the increasing antibiotic resistance of pathogenic bacteria [2]. This increase in antibiotic resistance can lead to experimental prescription of multiple antibiotics by physicians, thus perpetuating a vicious circle. The results of this study indicated that despite proven resistance to particular types of antibiotics in the microorganisms causing infection, the same antibiotic continued to be prescribed. This indicates that irrational antibiotic prescription can select for antibiotic resistance, thus increasing mortality and morbidity in all wards of the hospital and even in society. In contrast, the control of antibiotic resistance not only helps prevent infection caused by multi-drug resistant (MDR) microorganisms, but it can also decrease the cost of treatment [19]. 

## Notes

### Acknowledgment 

This study was supported by a grant (241/M/91201) from the National Institute of Health Research, the Islamic Republic of Iran, Tehran, Iran.

### Competing interests

The authors declare that they have no competing interests.

## Figures and Tables

**Table 1:**
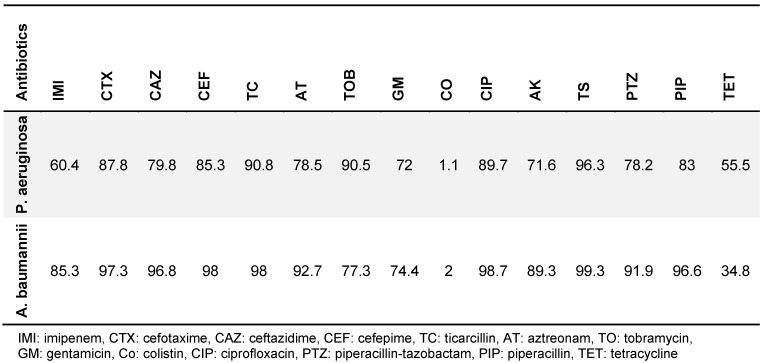
*A. baumanii* and *P. aeruginosa* resistance to various antibiotics in %

**Table 2:**
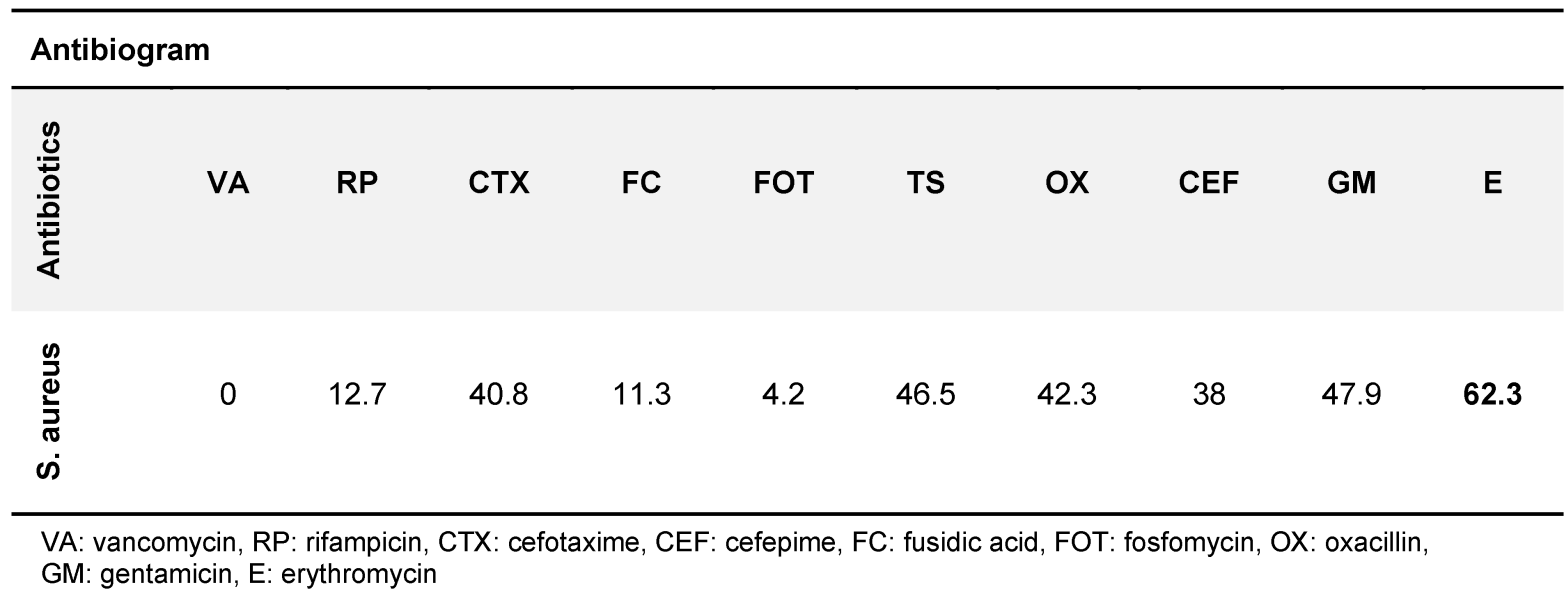
*S. aureus’* resistance to various antibiotics in %

**Table 3:**
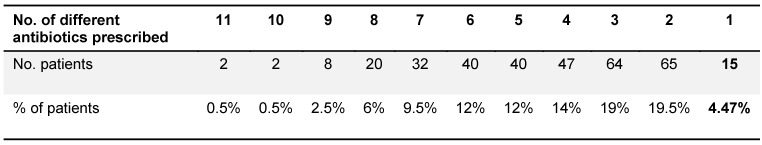
Number of different antibiotics prescribed and number and percent of patients in each group

**Table 4:**
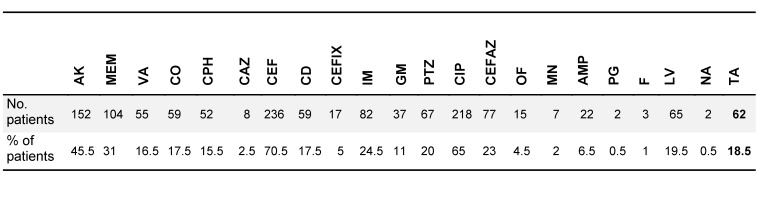
Number and percentage of patients prescribed different types of antibiotics

**Table 5:**
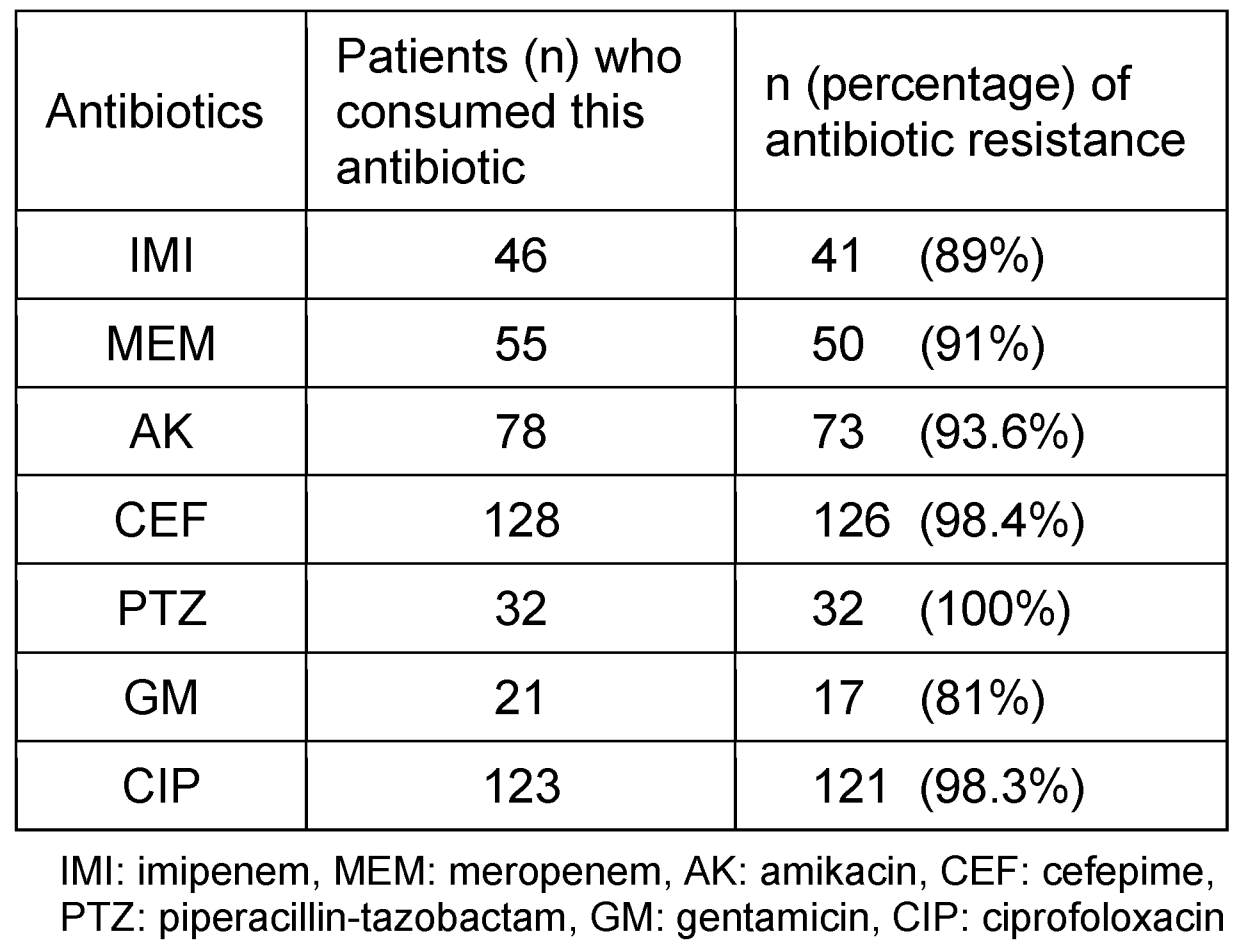
Antibiotic resistance according to antibiotic consumption
